# Vaccination in the Light of the Royal British Commission

**Published:** 1899-12

**Authors:** M. R. Leverson

**Affiliations:** Fort Hamilton, L. I., N. Y.


### VACCINATION IN THE LIGHT OF THE ROYAL BRITISH COMMISSION.

M. R. Leverson, M. D., Fort Hamilton, L. I., N. Y.

(Continued from Nov. No., page 496.)

Dr. Creigton in answer to Mr. Meadows White (Q. 5,047), gives the cases of Thornton, of Stowe, in which the variolous test failed. These cases were published by Dr. Beddoes, of Bristol, in his *Contributions to Physical and Medical Knowledge* in 1799. Mr. Savory, at Qs. 5,050-1-2, tried hard to make excuses for these failures on the ground that at that early stage it was natural that errors as to the time of taking the lymph, etc., should be made, etc.

Dr. Creighton disposes very conclusively of the stories of the opposition against which Jenner contended, showing on the contrary an anxiety to accept it, and that it was not until 1804, that opposition to it really commenced.

At Q. 5,068 he deals with Mr. Fry's experiments, saying that the inoculation with small-pox of milkers who had had the cow-pox produced no constitutional effect in thirty or forty cases. "Nor any greater degree of local inflammation than it would have done in the arm of a person who had before gone through the small-pox." But that was the same phrase with which Jenner had waived aside Shorter's cases in which variola unquestionably appeared. Then (Q. 5,074) we have the cases of the Coborne family in which two children and a lad in the employment of Mr. Coborne were vaccinated, were inoculated with small-pox, and all developed both the local pustule and a general eruption. Two other members of the family developed a local pustule. Mr. Savory at this explains that this local pustule was recognized by the observer as not being small-pox.*

---

*In my as yet unpublished work on the pathology of vaccination, I have shown that the description given of the cow-pox in these two cases resembles the classical descriptions of the syphilitic chancre.—M. R. L.

Q. 5,089. Mr. Hutchinson quotes from Dr. Creighton's *Jenner and Vaccination*, page 226, where fourteen cases are mentioned quoted from Von Sömmering: "Fourteen children have been vaccinated, all of whom were inoculated with variola afterwards and not one of whom had any eruption."

Q. 5,092. "You say at page 227, the absence of the general eruption was therefore no marvel?" "I think the absence of the general eruption was no marvel where the small-pox matter was inserted soon after the cox-pox, which was the case in Von Sömmering's cases, as it was certainly the case in the great body of variolous tests which were officially communicated to the Prussian Medical Department; in them the test was tried on the eighth or tenth day, and I assume it was about the same in Von Sömmering's experiments. I say the absence of the general eruption was no marvel, for this reason. The effect of cow-pox in the earlier removes from the cow was to cause a considerable swelling of the nearest absorbent glands, just as it used to do in the milkers, in whom it produced a painful affection in the glands of the armpits, which caused them to go about with their shoulders raised so the people knew at a glance they were suffering from cow-pox. * * * From that I conclude that another substance inoculated into the skin at or near the same parts, as it usually was, had not a good chance of being absorbed into the circulation, because it followed upon an acute inflammation of the lympathic glands which should have absorbed it.

Q. 5,094. "You admit, do you not, that the testimony is conclusive, that in those fourteen cases it did in some way prevent successful variolation? I should not confine myself to one thing as restricting the amount of small-pox produced by the test. I have already expressed the opinion that the mode of small-pox inoculation used in those years was chosen deliberatively for its limited effects."

Q. 5,097. (Dr. Collins). "You admit a convalescence prophylaxy, a protection lasting during the convalescence, or to put it in other words, do you explain those cases by a protective influence or an inhibitive influence being operative in the system or in the lymphatics upon the variola poison by virtue of the antecedent vaccine? I think I stated that the obstruction, so far as there was an obstruction, was of a mechanical nature."

Q. 5,111. Dr. Creighton then spoke of what he proposes to call the epidemic test; that is to say, small-pox broke out and a certain number of cases occurred among the vaccinated. Thereupon Jenner and his supporters endeavored to explain these cases away by asserting that the vaccinations had been spurious. It was a very elastic term and appears to have been applied co-extensively with occurrence of small-pox after vaccination.

The same plea was used in France and Germany as well as in England.

Q. 5,112. Historically it was a question of absolute protection or none, the plea of partial protection was put forward at a much later date. He refuses (Q. 5,115) to endorse a suggestion of Sir James Paget that Jenner and his supporters may have been mistaken. The middle course of asserting that although vaccine does not protect from small-pox, yet it determines a milder form of small-pox was adopted later (Q. 5,117). They said it modified the kind of small-pox which ensued; but that was a rather late development.

Q. 5,119. "I do not quite understand how that becomes important? If you ask me my opinion, it seems to me the conclusion ought to have been that the cow-pox had no relation to small-pox."

Qs. 5,120-25. Efforts are made to entrap Dr. Creighton into admissions with regard to statistical evidence, but he declines to go out of the pathological and historical lines on which he had come to testify. At last the chairman at Q. 5,125, asks, "Do you think upon these early facts to which you allude that the conclusion would be justified that vaccination was an illusion without an examination of the facts which experience has furnished us with during the many years that vaccination has been in operation?" To which he replies, "No, I do not; and, personally, before I came to a conclusion in the matter, I examined the statistical evidence also."

Q. 5,126. "And as I understand it, drew your conclusion from the early history of vaccination, that it is a delusion and an imposture which has been fostered by the medical profession, and which has no other foundation? I suppose the refrence is to page 352, where I say: 'The anti-vaccinists are those who have found some motive for scrutinizing the evidence, generally the very human motive of vaccinal injuries or fatalities in their own families or in those of their neighbors. Whatever their motives they have scutinized the evidence to some purpose; they have mastered nearly the whole case, they have knocked the bottom out of the grotesque superstition.' "

Q. 5,127. "I would not have said that much if I had not made a careful examination of the statistical evidence, and allowed it to weigh with me along with the pathological and historical facts to which I have been calling attention."

Q. 5,128. (Dr. Bristow.) "Then you agree with Mr. White? I do, as you ask me."

Q. 5,129. Dr. Creighton next took up the supposed scientific connection between cow-pox and small-pox as sought to be proved by the experiments of Thiele, Ceely, and others, down to Dr. Voigt, of Hamburg. He goes into details with regard to the last of these. The attempt as to all was to inoculate small-pox on the cow. The result was that when a sore was produced on the cow and the matter from it inoculated on man it produced small-pox. But Dr. Voigt's experiments have been cited to prove that small-pox after its inoculation upon a calf is transformed into cow-pox, but Dr. Creighton reports them in full, and the mere statement serves to show that all his simple small-pox inoculations produced small-pox; but in a fourth experiment he inoculated small-pox at five shallow incisions from a discrete case on the shaven surface of the left hypogastrium, and at the same time he inoculated cow-pox matter at a spot, as he says, sufficiently remote from the other; but does not say where the spot was. Four out of his five small-pox insertions came to nothing, the fifth became a large yellowish-white vesicle. The cow-pox inoculation also produced vesicles from which lymph was taken. A number of children were inoculated with the lymph. Then from the large, pearly vesicle he inoculated a calf at twelve incisions and all produced a crop of vaccine vesicles, and from that calf he inoculated a child which had axiliary swelling upon the ninth day and six discrete nodules under the skin for several weeks after. A second child had an areola and pain in the axilla. A third child, inoculated at eight places, had all by the fourteenth day turned to large gray ulcers surrounded by erysipelas. Dr. Voigt discusses the question whether he had not mixed up on the calf's hypogastrium the small-pox inoculation and the cow-pox inoculations.*

The commisson press Dr. Creighton to give his view as to that, but he resisted all their pressure untill finally (Q. 5,137) Dr. Collins says: "Do you think it fair to say that the experiment of Dr. Voigt served to illustrate that small-pox inoculated upon the cow would give cow-pox?" To which he replies, "I think the evidence is insufficient for that conclusion;" and again (Q. 5,140) he knows of no experiments which are unambiguous, which demonstrate the possibility of converting small-pox into cow-pox by inoculating a cow.

---

* Dr. Voigt resolves the question in favor of the lymph. It is evident from his argument that he looked upon the cow's body as something in the light of a building having different chambers, into one of which you might place something which will have no connection with any other chamber. This strange misconception goes through much of the pro-vaccinist literature.—[*Ed.*]

In repy to Sir James Paget (Q. 5,142), he says that he believes Ceely gave small-pox to the children he vaccinated; that is to say, small-pox of the inoculated sort.

Q. 5,143. That this was carried on through several removes, as has been frequently done in the last century, and that in all the successive vaccinations Ceely carried on from child to child and arm to arm, he really gave them small-pox, that if it was small-pox in the first, he does not know what else it could have become in the other cases, and that down to the last case which Ceely described of vaccination with this matter was nothing else than ordinary small-pox as inoculated.

Q. 5,149. Cow-pox and small-pox are different in local effects and in the epidemiological facts and in their clinical history.

Q. 5,151. Sir James Paget calls attention to Ceely's great care and accurate observation, and asks Dr. Creighton if he could have done wrongly in regarding them as cases of vaccination, to which Dr. Creighton replies, "I think that nothing was impossible in the matter."

The chairman then (Q. 5,152) asks whether the fact that a most competent observer mistakes one for the other in a large number of cases does not prove that small-pox in one of its forms must be very similar to cow-pox; but Dr. Creighton shows that he was dominated by Jenner's "original idea," that cow-pox was small-pox of the cow. "He was under the influence of the idea that he was going to prove the identity of cow-pox and small-pox by this experiment on the heifer, when one would suppose that a very brief study of the epidemiological history of small-pox, and on the other hand, of the morbid anatomy of cow-pox, would have kept him right," and then, being still further pushed on the question, he says: (Q. 5,153) "They may have made such an impression upon the mind of Mr. Ceely according to the principle, *credo quia impossibile, which has a wide application to mysterious things!*"

Q. 5,154. (Dr. Bristow.) "But you have no ground for believing, have you, that there is any anatomical difference between cow-pox and small-pox?" *"I have, indeed.* I so stated at the beginning of my evidence."

[We have here again a striking illustration of a *preconceived idea,* as Dr. Creighton so well shows. Another illustration has very recently come under the observation of the editor of these abstracts. A physician in large practice in Mississippi, where a small-pox scare prevails at the present time, was asked by a member of the bar to criticise the pathological table of small-pox, cow-pox, and syphilis, familiar to the readers of THE HOMŒOPATHIC PHYSICIAN. He told him that he found no fault with the table, which had, no doubt, been correctly compiled from the books, but that cow-pox and small-pox were the same disease although the symptoms might be different, and that cow-pox and syphilis were distinct although many of their symptoms might be the same!—M. R. L.]

